# Case Report: A rare case of symptomatic bradycardia secondary to aripiprazole in a patient with bipolar disorder type I

**DOI:** 10.12688/f1000research.126979.1

**Published:** 2022-10-31

**Authors:** Mailing Flores Chang, Nehemias Guevara, Marlon Argueta, Yomary Jimenez, Adler Vitaly, Salim Baghdadi

**Affiliations:** 1 Department of Medicine, Internal Medicine, St. Barnabas Health System, The Bronx, New York, 10487, USA; 2 Department of Medicine, Internal Medicine, Division of Cardiology, St. Barnabas Hospital Health System, The Bronx, New York, 10457, USA

**Keywords:** Bradycardia, atypical antipsychotic, Aripiprazole, Bipolar disorder.

## Abstract

It is well known that typical antipsychotic drugs have been implicated as a cause of ventricular arrhythmias and cardiac arrest; studies have shown that conventional antipsychotics increase the risk of hospitalization for ventricular arrhythmias or cardiac arrest nearly 2-fold. However, atypical antipsychotics are not associated with an increased risk of hospitalization for ventricular arrhythmias or cardiac arrest. The use of atypical antipsychotics increased since they were first discovered and now are the mainstay of treatment, but with their broad use, heart effects have been documented, such as prolonged QT interval. Clozapine has been linked to severe cardiac problems, and risperidone has been linked to an increased risk of ventricular arrhythmias and cardiac arrest. We present a case of a patient with bipolar disorder who presented with symptomatic bradycardia secondary to aripiprazole.

## Introduction

Several medication classes benefit psychotic disorders, such as schizophrenia and bipolar disorder. Over the years, atypical antipsychotics have been deemed first-line treatment due to their beneficial effects on overall mood and cognition. However, their detrimental effects, such as obesity, diabetes mellitus, and dyslipidemia, should not be ignored. ^1^ These medications also have increased cardiovascular side effects, including QTc prolongation, torsade de Pointes (TdP), sudden cardiac death, myocarditis, and cardiomyopathy. ^2^^,^^3^ In addition, patients with psychotic disorders already have an increased risk of cardiovascular mortality. ^4^

The complexity of the mechanism of action of atypical antipsychotics is broad and still under active investigation ^5^^,^^6^; however, direct cardiovascular risks overweight their benefits. ^7^

Although there is extensive evidence of the detrimental effects of atypical antipsychotics on cardiovascular health, there is not enough evidence of the effect of aripiprazole on heart rate (HR) and how it causes bradycardia. To the best of our knowledge, there are only two cases of aripiprazole related to bradycardia/syncope. ^4^^,^^8^

We present a case of a patient with bipolar disorder with aripiprazole-induced symptomatic bradycardia that did not respond to standard treatment such as atropine and resolved with medication discontinuation.

## Case report

A 61-year-old Latin male, unemployed at the moment, with a medical history of bipolar disorder type I diagnosed one year ago, recently started on aripiprazole. The patient did not have any further relevant family or social history. He presented to the emergency department (ED) complaining of intermittent episodes of dizziness initiated after his second dose of intramuscular (IM) aripiprazole which had worsened three days prior to admission. He denied any use of drugs or other medications. His last aripiprazole dose was two weeks before admission.

**Figure 1.**
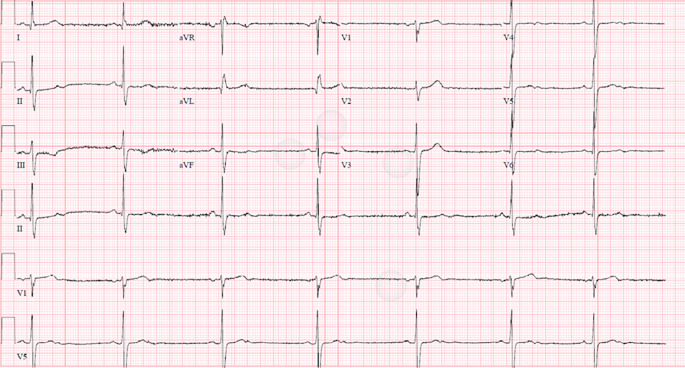
Electrocardiogram.

Upon arrival at the ED, the patient was found to be bradycardic (44 beats/minute), normotensive, and had pulse oximetry of 98% on room oxygen. An electrocardiogram showed sinus bradycardia of 44 bpm with QT of 410 msec ( Figure 1). Emergent management with atropine 0.5 mg IV was started which increased the heart rate to 50 beats/minute; however, the patient’s intermittent dizziness persisted.

**Figure 2.**
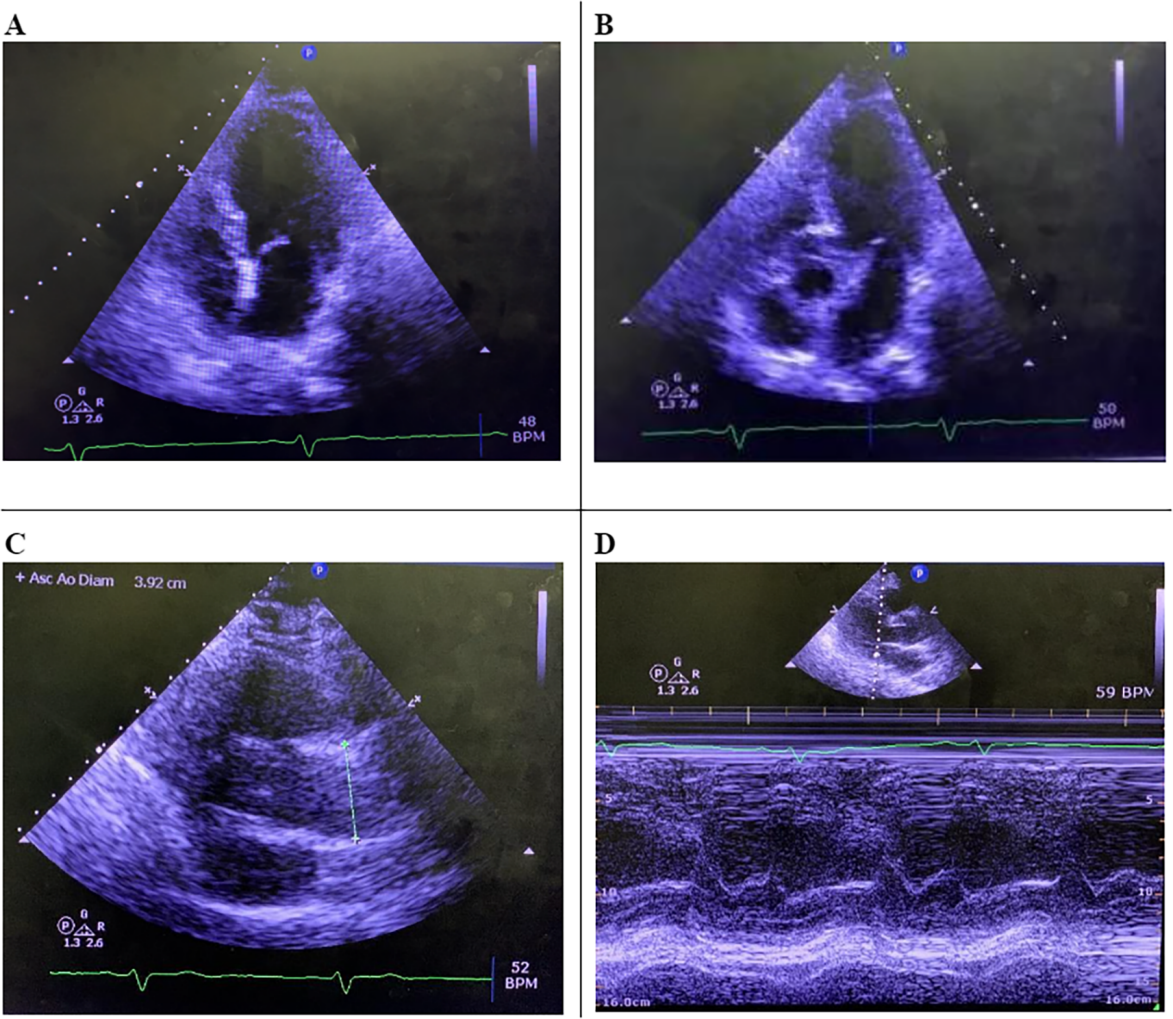
Echocardiogram – A: Four chamber projection, B: Five chamber projection, C: This view showing aortic root diameter, D: Mitral valve – M mode.

**Figure 3.**
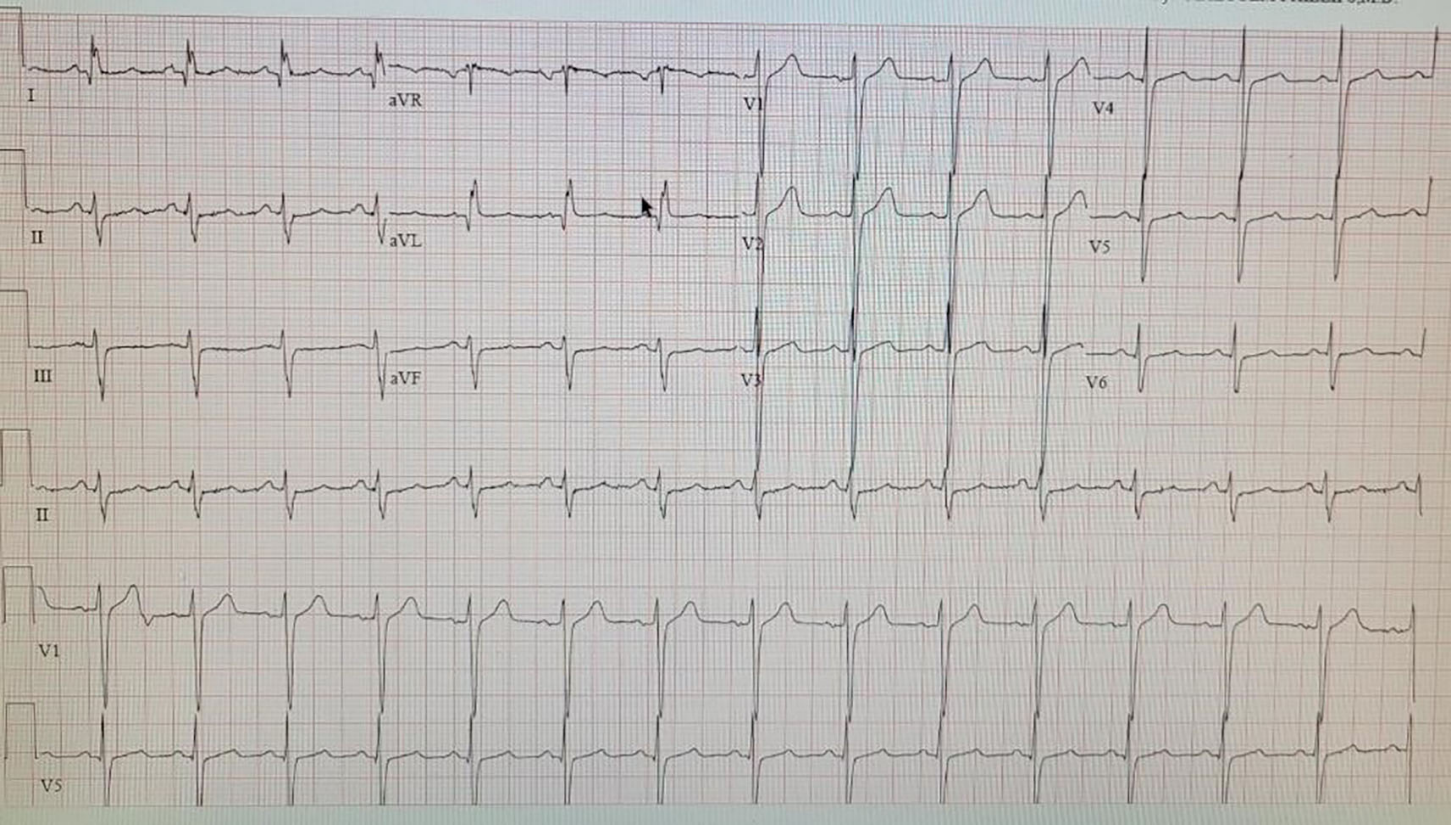
Follow-up electrocardiogram.

Initial hematologic and chemistry blood work was unremarkable ( Table 1). The COVID-19 antigen test was negative. Cardiology evaluation was unremarkable. The stress test showed a peak heart rate of 144 (90 % of the maximum predicted for his age) and was negative for any ischemia. A 24-hour Holter and echocardiogram were within normal limits ( Figure 2). At this point, after ruling out the most frequent causes of bradycardia, such as ischemia, medication reconciliation showed a possible association between atypical antipsychotics and previous cases reported of bradycardia. Therefore, a psychiatric evaluation was requested; psychiatry saw the patient 4 days after admission and suggested discontinuation of aripiprazole. Naranjo score calculations showed a score of 9, which put the patient as probable his symptoms were caused by an adverse effect of aripiprazole. After 4 days of discontinuation of aripiprazole, patient’s heart rate improved to 55 beats per minute, therefore, a resolution of symptoms. The patient was discharged on risperidone 1 mg twice a day and sodium valproate 500 mg twice a day. After discharge, patient was seen for the first time as an outpatient 4 months later at the cardiology clinic and he has not reported similar symptoms; furthermore, a follow-up EKG showed a complete resolution of bradycardia ( Figure 3). Although aripiprazole was suspected to be the cause at the time of hospitalization and discharge due to the patient’s improvement, this was not confirmed until the subsequent follow-ups which showed a complete resolution of symptoms.

**Table 1.** Laboratory data.

Variable	On admission	Reference range
White cell count	6.0	4.2 – 9.1 10*3/uL
Neutrophils	52.6%	34.0 – 67.9%
Lymphocytes	37.4	21.8 – 53.1 %
Monocytes	9.0	5.3 – 12.2 %
Eosinophils	0.5	0.8 – 7.0%
Hemoglobin	14.5	13.7 – 17.5 gm/dL
Hematocrit	44.0	40.1 – 51.0 %
Platelet count	199	150 – 450 10*3/uL
MCV	92.1	79.0 – 92.2 fL
MCH	30.3	25.7 – 32.2 pg
MCHC	33.0	32.3 – 36.5 gm/dL
Sodium	139	135 – 145 mEq/L
Potassium	4.7	3.5 – 5.3 mEq/L
Chloride	104	96 – 108 mEq/L
Glucose	93	70 – 99 mg/dL
Calcium	9.4	9.2 – 11.0 mg/dL
Creatinine	0.9	0.6 – 1.2 mg/dL
ALT	28	4 – 36 IU/L
AST	22	8 – 33 IU/L
Bilirubin Total	0.7	0.1 – 1.2 mg/dL
TSH	2.36	0.34 – 5.60 u [IU]/mL
Troponin	0.01	0.00 – 0.48 ng/mL

## Discussion

Atypical antipsychotic drugs came out in the 1990s; we currently have clozapine, risperidone, olanzapine, quetiapine, ziprasidone, and aripiprazole.

They have been associated with multiple side effects, such as obesity, diabetes mellitus, and dyslipidemia; therefore, cardiovascular risk increases when taking these medications. ^2^^,^^3^

Specific cardiovascular side effects include orthostatic hypotension and reflex tachycardia; these have been linked to the antagonization of α1 receptors.

However, more serious cardiac side effects also have been reported, such as sudden cardiac death due to anticholinergic activity; ventricular tachycardia, torsades de pointes (TdP), delayed cardiac repolarization, myocarditis, myocardial infarction, and cardiomyopathy. ^9^^–^^11^

Aripiprazole was approved in 2002 for schizophrenia and shortly after for bipolar disorder I; benefits such as less propensity for weight gain, favorable metabolic profile, and no association with hyperprolactinemia make it a good option. ^8^ Furthermore, multiple clinical trials have shown safety profiles in acute and chronic symptoms of schizophrenia and bipolar disorder I. The most common share adverse events were headache, agitation, insomnia, anxiety, akathisia, and somnolence. An increased level of prolactin was also reported. ^5^^,^^7^^,^^12^^–^^14^

Potkin *et al*. (2003) reported small changes in QTc but these were not significant; however, there is evidence that atypical antipsychotics can cause prolongation of the QT, therefore, inducing ventricular tachycardia and torsades de pointes. ^5^

Bradycardia has been reported after the initiation of atypical antipsychotics, ^15^^,^^16^ but we found only two cases where aripiprazole was associated as the causative drug of symptomatic bradycardia. ^4^^,^^8^

The mechanism of action for which atypical antipsychotics could cause bradycardia has been related to the effects on 5-HT1A (5-beta hydroxytryptamine receptor 1) activation with upregulation of α2-adrenoreceptors in the brainstem. ^8^^,^^15^^,^^17^^,^^18^

The complex signaling and the role played by the sympathetic and parasympathetic nervous systems, as well as serotonin pathways in the heart, is complex and is the reason behind this type of medication can have an effect on the heart. ^18^^–^^20^

Aripiprazole is a partial dopamine agonist with substantial binding affinity for the serotonin 5HT2A (short for 5-hydroxy-tryptamine subtype 2 A) receptor; it has a broad spectrum receptors interaction as mentioned by *Shapiro et al.* ^6^ with high affinity for h5-HT(2B) (5-hydroxytryptamine receptors), hD(2L)-, and hD(3)-dopamine receptors. Furthermore, an affinity for several other 5-HT receptors such as (5-HT(1A), 5-HT(2A), 5-HT(7)), as well as alpha(1A)-adrenergic and hH(1)-histamine receptors.

Aripiprazole has less affinity (30-200 nM) for other G protein-coupled receptors, including the 5-HT(1D), 5-HT(2C), alpha(1B)-, alpha(2A)-, alpha(2B)-, alpha(2C)-, beta(1)-, and beta (2)-adrenergic, and H(3)-histamine receptors. ^6^^,^^21^

Our patient arrived with symptomatic bradycardia negative normal thyroid gland function test (TSH: 2.36). An echocardiogram showed borderline left ventricular hypertrophy and normal left ventricular wall motion and ejection fraction, with a normal stress test.

Typical medications that are associated with bradycardia were ruled out, such as beta-blockers.

Aripiprazole 400 mg IM monthly injections and 5 mg oral daily two months prior to his hospitalization, with the last dose two weeks prior to admission, make this association possible. Furthermore, Naranjo Adverse Drug Reaction Probability Scale was calculated and was 9. ^22^

As per the literature review, in all the cases reported where the bradycardia cause was atypical antipsychotics ^6^^,^^23^ all symptoms were reversible after stopping the medication, and our case was not the exception.

He has been following up in our clinic without the persistence of his symptoms and normal EKGs.

## Conclusion

Multiple cardiovascular risk factors have been associated with the use of antipsychotic medications, such as ventricular tachycardia, TdP, delayed cardiac repolarization, myocarditis, myocardial infarction, and cardiomyopathy. However, there is limited evidence regarding antipsychotic-induced bradycardia, specifically aripiprazole-related. As observed in our case, the patient with bipolar disorder treated who was with aripiprazole developed symptomatic bradycardia which was induced by the use of this medication. This was proven after extensive cardiac workup that resulted in negatives for other common causes such as ischemia; furthermore, symptoms resolved after discontinuation of the medication.

Although we could prove with our case that aripiprazole can cause bradycardia, further studies with more data are needed to establish a clear relationship between this medication and bradycardia.

## Patient perspective

The patient states he started to feel unwell after initiating the new treatment but never associated his symptoms with the new medication. Upon discharge, he understood what symptom management he had received and received a full explanation of the new treatment's side effects.

## Consent for publication

Informed written consent was obtained from the patient for the publication of this case report and the accompanying images.

## Authors' contributions

• All authors have contributed equally to this case report.

## Data Availability

All data underlying the results are available as part of the article and no additional source data are required.
