# Supplementary material for: Case Report: A rare case of symptomatic bradycardia secondary to aripiprazole in a patient with bipolar disorder type I
Source: F1000Res. 2022 Oct 31;11:1233. [Version 1] doi: 10.12688/f1000research.126979.1 (PMC10818102; doi:10.12688/f1000research.126979.1)
Supplement: Peer review history

# Reviewer report: Reviewer response for version 1

**DOI:** 10.5256/f1000research.139439.r227261

**Published:** 2024-01-26

**Authors:** Barun Kumar

**Affiliations:** 1 Department of Cardiology, All India Institute of Medical Sciences, Rishikesh, Uttarakhand, India

Well-written case report. Although a rare side effect, timely recognition is important as at this age one may confuse this with sick sinus syndrome and may implant a pacemaker. As the patient recovered and didn't show bradycardia, it proves that the drug was the culprit. A longer follow up will further support the authors' observations. Congratulations.

Are enough details provided of any physical examination and diagnostic tests, treatment given and outcomes?

Yes

Is the case presented with sufficient detail to be useful for other practitioners?

Yes

Is sufficient discussion included of the importance of the findings and their relevance to future understanding of disease processes, diagnosis or treatment?

Yes

Is the background of the case’s history and progression described in sufficient detail?

Yes

Reviewer Expertise:

Cardiology, coronary artery disease, Angioplasty & Stenting.

I confirm that I have read this submission and believe that I have an appropriate level of expertise to confirm that it is of an acceptable scientific standard.

# Reviewer report: Reviewer response for version 1

**DOI:** 10.5256/f1000research.139439.r227264

**Published:** 2023-12-18

**Authors:** Liliang Li

**Affiliations:** 1 School of Basic Medical Sciences, Fudan University, Shanghai, China

Authors described a rare case who presented symptomatic bradycardia secondary to aripiprazole, but recovered after replacement with risperidone treatment. This is an interesting case that would arouse attention to clinical treatment. I have the following suggestions:

 1. Authors need to state the metabolic parameters and BMI since these are very important risk factors for cardiovascular changes.

 2. Discussion section is not well-organized and did not cover the recent research progress. First, authors should delete some redundant and repeated statements. Second, since aripiprazole also belongs to atypical antipsychotics, authors may need to discuss whether the bradycardia is drug-dependent? I suggest authors refer to two recent literatures (PMID:34733639 and 35739093).

 3. Authors may revise their language.

Are enough details provided of any physical examination and diagnostic tests, treatment given and outcomes?

Yes

Is the case presented with sufficient detail to be useful for other practitioners?

Yes

Is sufficient discussion included of the importance of the findings and their relevance to future understanding of disease processes, diagnosis or treatment?

No

Is the background of the case’s history and progression described in sufficient detail?

Yes

Reviewer Expertise:

Antipsychotics cardiotoxicity

I confirm that I have read this submission and believe that I have an appropriate level of expertise to confirm that it is of an acceptable scientific standard, however I have significant reservations, as outlined above.

## References

[1] : Antipsychotics cardiotoxicity: What's known and what's next. *World J Psychiatry* .2021;11(10) : 10.5498/wjp.v11.i10.736 736-753 10.5498/wjp.v11.i10.736 34733639 PMC8546771

[2] : CB1R-stabilized NLRP3 inflammasome drives antipsychotics cardiotoxicity. *Signal Transduct Target Ther* .2022;7(1) : 10.1038/s41392-022-01018-7 190 10.1038/s41392-022-01018-7 35739093 PMC9225989

# Reviewer report: Reviewer response for version 1

**DOI:** 10.5256/f1000research.139439.r154570

**Published:** 2022-12-06

**Authors:** Juan Manuel Muñoz Moreno

**Affiliations:** 1 Department of Cardiology, Hospital Nacional Edgardo Rebagliati Martins, Lima, Peru

Flores Chang, *et al.,* describe an interesting case of symptomatic bradycardia associated with the use of the atypical antipsychotic, aripiprazole.

 I would like to add a few minor observations:

- An electrocardiogram showed sinus bradycardia of 44 bpm with QT of 410 msec: describe the QT interval corrected for HR, and note that it was in the normal range.
- Our patient arrived with symptomatic bradycardia negative normal thyroid gland function test (TSH: 2.36). An echocardiogram showed borderline left ventricular hypertrophy and normal left ventricular wall motion and ejection fraction, with a normal stress test. Typical medications that are associated with bradycardia were ruled out, such as beta-blockers.
  -- > It could be written as: Our patient arrived with symptomatic bradycardia, and alternative causes such as the use of beta-blockers, alterations in thyroid hormones and myocardial ischemia were ruled out.

Are enough details provided of any physical examination and diagnostic tests, treatment given and outcomes?

Yes

Is the case presented with sufficient detail to be useful for other practitioners?

Yes

Is sufficient discussion included of the importance of the findings and their relevance to future understanding of disease processes, diagnosis or treatment?

Yes

Is the background of the case’s history and progression described in sufficient detail?

Yes

Reviewer Expertise:

Bradyarrhythmias, antiarrhythmic, heart disease

I confirm that I have read this submission and believe that I have an appropriate level of expertise to confirm that it is of an acceptable scientific standard.
